# Ultrastructure change and transcriptome analysis of GA3 treatment on seed germination of moso bamboo(*Phyllostachys edulis*)

**DOI:** 10.1080/15592324.2022.2091305

**Published:** 2022-07-07

**Authors:** Juan Li, Yucong Bai, Yali Xie, Jian Gao

**Affiliations:** Gene Science and Gene Industrialization Institution, International center for Bamboo and Rattan, Beijing, China

**Keywords:** Moso bamboo, seed germination, GA_3_, respiration rate, TEM, transcriptome analysis, key genes

## Abstract

Exploring the mechanism of Gibberellic acid (GA_3_) treatment on seed germination of moso bamboo can lay a foundation for its future breeding and research. In this study, the germination-related indicators (germination rate, germination potential, vigor index, respiration rate) with different content of GA_3_ treatment were measured, and the ultrastructure of moso bamboo seeds treated with low and high GA_3_ concentrations was observed during the germination process. In addition, the transcriptome data of the germination seeds, with and without GA_3_ treatment were analyzed. The results showed that the low GA_3_ concentration (10 mol/L) increased the germination rate, germination potential, vigor index and respiration rate, thus promoting the germination of moso bamboo seeds, but a high concentration of GA_3_ (50 mol/L) inhibited the seed germination. The low GA_3_ concentration accelerated the decomposition of starch and fat and promoted the vacuole formation of cells, but the high GA_3_ concentration damaged organelles and increased the endocytosis of cells. Compared with untreated moso bamboo seeds, the seeds had fewer genes expressed after GA_3_ treatment. Starch and carbon metabolism play a very important role in seed development and embryo viability, whether the seed is treated with GA_3_ or not. After hormone treatment, GID1 and DELLA-related genes homologous to rice genes is not expressed, but the expression of PIF4, PIF5, GA_3_ *ox*2, GA_2_*oxs*, etc., were up-regulated.

## 1. Introduction

Gibberellic acid (GA_3_) is a common plant growth regulator that plays an important role in seed dormancy and germination.^1^ The low GA_3_ concentrations can break seed dormancy and improve seed germination rate and germination potential.^2–5^ The GA_3_, with appropriate concentrations, can enhance enzyme activity, promote the decomposition of storage materials, increase sesame yield and oil content, stimulate embryo growth, and boost the breakthrough of radicle through seed coat, thus promoting seed germination.^6–10^

Gibberellic acid (GA) regulates the transcription aPPMnd translation of germination-related genes through the signal transduction pathway, thus regulating seed dormancy and germination. Gibberellin insensitive dwarf 1(GID 1), GA-Respond MYB (GAMYB), and DELLA protein genes are the key genes in the GA signal transduction pathway. The GID 1 is the receptor protein of GA;^11,12^ GAMYB is the trans-acting factor^13^and DELLA protein is a negative regulator of the GA signal transduction pathway. The five DELLA proteins in Arabidopsis, GA INSENSITIVE (GAI), REPRESSOR OF ga1-3 (RGA), RGA-LIKE 1 (RGL1), RGL2 and RGL3, are associated with seed germination.^11^ It showed that the RGA-LIKE3 (RGL3), is important for oil and seed storage protein accumulation.^14^

It is essential to understand the viability and ultrastructure of seeds. Some changes in the germination process of recalcitrant seeds are related to GA treatment, such as the formation of vacuoles, increased mitochondria and the development of endoplasmic-reticulum.^15,16^However, the microstructure changes during the germination of moso bamboo seeds after hormone treatment has not been investigated.

Moso bamboo (*Phyllostachys edulis*) (Poaceae, Bambusoideae) is one of the most abundant bamboo species in China and has important social, economic, and ecological functions. It is characterized by long-term asexual reproduction and flowering uncertainty in time and space. In addition, it blooms once in a lifetime and dies thereafter.^17,18^ Authors^19,20^ have reported that the seeds of moso bamboo have irregular germination periods and short storage time (less than one year under natural conditions). This greatly restricts the germplasm preservation and scientific production and selection of bamboo seeds and also impedes their multi-generation genetic improvement. The vitality of aging moso bamboo seeds was improved after PEG and GA_3_ initiation treatment under suitable conditions, and the severe aging degree of seeds indicates the more significant enhancement in seed vigor.^21^ However, the optimal concentration and mechanism of GA_3_ for the germination of moso bamboo seeds are still unknown.

Although the dormancy moso bamboo seeds can be broken by hormone treatment, the molecular mechanism of seed development after hormone treatment is still unclear. In this study, the respiration rate, germination rate, germination potential and vigor index of moso bamboo seeds, treated with different concentrations of GA_3_, were studied. In addition, the ultrastructure of endosperm, with exogenous GA_3_ treatment, during seed germination was observed, the transcriptome data in moso bamboo seeds treated with GA_3_ was analyzed. Our findings will serve as a public information resource for future studies on the germination of moso bamboo seeds.

## 2. Materials and methods

### 2.1. Plant materials

Seeds of moso bamboo were obtained in Dajing County, Guilin (Longitude: E110°17’–E110°47’; Latitude: N25°04’–N25°48’, altitude: 800–900 m) in Guangxi Zhuang Autonomous Region in August 2017. The seeds were collected from the flowering bamboo forest (area about 6000 m^2^). The bamboo stems were green and without leaves. The bamboo forest all died the next year after collection. Five kilogram seeds (500 g per plant, 50 cm spacing) of ten plants were collected. Seeds were mixed fully and transported to the laboratory for test several days later.

### 2.2. Seed treatment and seed germination experiment

Fresh seeds of moso bamboo were used for germination tests, which were repeated three times. Seed coats were peeled off, a total of 500 were soaked in cold water for 48 h, and then the seeds were washed with detergent and running water for 0.5–1 h and then with distilled water before placing them on an ultra-clean table. The seeds were soaked for 4–6 h in 0.5%–0.8% formaldehyde, shocked several times, rinsed with sterile water four times after disinfection, and then placed on two sheets of moistened filter paper in 9-cm Petri dishes. Ten milliliter of gibberellin solution at five gradients (10 mmol/L, 20 mmol/L,30 mmol/L,40 mmol/L, and 50 mmol/L) was injected into the petri dish with seeds.^22^ Petri dishes were placed in a dark room at 25°C. Daily germination observations were photographically recorded. Germination can be defined as the phase from seed sowing until the emergence of a vigorous seedling, four different types were distinguished: primary stage (Stage II), middle stage (Stage III), final stage (Stage IV), and fresh seeds soaked in cold water for 48 h that nonetheless did not germinate were used as Stage I seeds.^23^ A total of 50 seeds of each type were placed in an ultra-low temperature freezer for RNA (Ribonucleic Acid) extraction and enzyme determination. Then five seeds of each type were assessed for respiratory rate: These seeds were fixed in FAA (Federal Aviation Administration) 38% formaldehyde: glacial acetic acid: 70% alcohol methanol (1:1:18, v/v) for at least 48 h for determination of TEM (Transmission electron microscope).Germination rate, germination potential, germination index were calculated.

### 2.3. Gene expression analysis of RNA-Seq data

Total RNA from different stage germination seed of treated with GA_3_ and untreated was isolated using TRIzol reagent (Invitrogen, CA, USA).The RNA quality was evaluated by electrophoresis through a 1% agarose gel, and the RNA concentration was determined by absorbance at 260 nm using a Nanodrop spectrophotometer (Nanodrop Technologies, USA). A total amount of 1 μg RNA per sample was used as input material for the RNA sample preparations. Sequencing libraries were generated using the NEBNext UltraTM RNA Library Prep Kit for Illumina (NEB, USA). High-throughput RNA-Seq was performed on an Illumina HiSeq 2000. Two biological replicates at each stage of each treatment were used for the analysis. The raw data generated by Illumina sequencing were deposited in NCBI SRA under accession number PRJNA627339 and PRJNA842835.

KEGG (Kyoto Encyclopedia of Genes and Genomes) classification of DEGs were obtained. The identification of significant DGE (Polyethylene Glycol) models of treated with GA_3_ and untreated sample groups was performed with the DESeq R package (1.10.1). Genes with an adjusted P-value < 0.05 found by DESeq were considered differentially expressed. Gene ontology (GO) enrichment analysis of the differentially expressed genes (DEGs) was implemented by the GO seq R packages based on Wallenius non-central hyper-geometric distribution, which can adjust for gene length bias in DEGs. TF (transcription factors) were extracted from the DEGs.

### 2.4. Gene network construction and visualization

The data were obtained from the transcriptome data of moso bamboo seeds treated with GA_3_ and untreated during germination. A total of 14 transcriptome data were used, include 13865 genes with FPKM (Fragments Per Kilobase of Exon Per Million Reads Mapped) >1 of GA_3_ treatment, and 8015 genes with FPKM >1 of no treatment, which were imported into WGCNA (Weighted Correlation Network Analysis) (v1.29) package used for co-expression network clustering analysis.^24^ Genes are presented in the module format. Modules had a power of 6, a minimum module size of 30, and a merge cut height of 0.20. The module was associated with each test sample according to its eigengene value. The hub genes and networks were visualized using Cytoscape.^25^

### 2.5. Gene expression analysis by qPCR

QRT-PCR (Quantitative Real-time Polymerase Chain Reaction) analysis was performed to estimate the validity of the transcriptome sequences. RNA samples used for qRT-PCR were identical to those used for the RNA-Seq. In total, twelve DEGs were selected, and gene-specific primers were designed using Primer Premier 6.0. Approximately 1 μg of DNase I-treated total RNA from 4 stages was converted into single-stranded cDNA using a PrimeScript RT reagent kit (Takara, Dalian, China). TIP41 was used as the inner reference gene. qRT-PCR was performed using SYBR® Premix Ex Taq™ II (Tli RNaseH Plus) (RR820b, TAKARA, DaLian, China) on a Roche LightCycler® 480 (Applied Biosystems, USA) with three technical replicates. Amplification reactions were initiated with a denaturing step (95°C for 30 min), followed by 40 cycles of denaturing (95°C for 5 s), annealing (60°C for 30s), and extension (72°C for 30s), for a total of 40 cycles.

### 2.6. Respiration rate analysis

The seed respiration rate was determined polarographically using a Clark-type oxygen electrode connected to a computer-operated Oxygraph control unit (Hansatech Instruments, Norfolk, UK). After washing in sterile distilled water, five seeds of each type (Stage I, Stage II, Stage III, Stage IV in different GA_3_ treatment) were placed in the respiration buffer (5 mL deionized water, pH 7.2) in the reaction cup of the instrument and read on the computer after 1 min. For all experiments, the temperature was maintained at 25°C. Respiration rates were expressed as nanometer of oxygen consumed per milliliter per minute. The experiment was performed three times.

### 2.7. Transmission electron microscopy analysis

The general procedures of preparing conventional samples for TEM, described previously, were followed with minor modifications.^26^ Briefly, middle areas of embryo sections (1 mm^3^) (StageI,StageII,StageIII,Stage IVin different GA_3_ treatment) were cut from seedlings (1-week old) and fixed immediately in 2.5% glutaraldehyde in PBS(Phosphate Buffer Saline) overnight at 4°C. The tissues were rinsed three times using PBS, post-fixed in 1% osmium tetroxide for 4 h, rinsed three times using PBS again, dehydrated in a graded acetone series, and embedded in SPI-PON 812 resin (SPI Supplies). For TEM, the ultrathin sections were contrasted with uranyl acetate and lead citrate and were observed directly under the TEM (HT7700, Japan) at 120 kV.

### 2.8. Statistical analyses

Excel 2007 and SPSS (Statistical Product and Service Solutions) 14.0 were used for statistical analysis, with one-way ANOVA (Analysis of Variance) and LSD (Least Significance DifferenceTest) for variance analysis and multiple comparisons (α = 0.05). The data in the tables and figures are expressed as mean value ± standard deviation.

## 3. Results

### 3.1. Germination indexes of moso bamboo seed under different GA_3_ treatments

**Figure 1.**
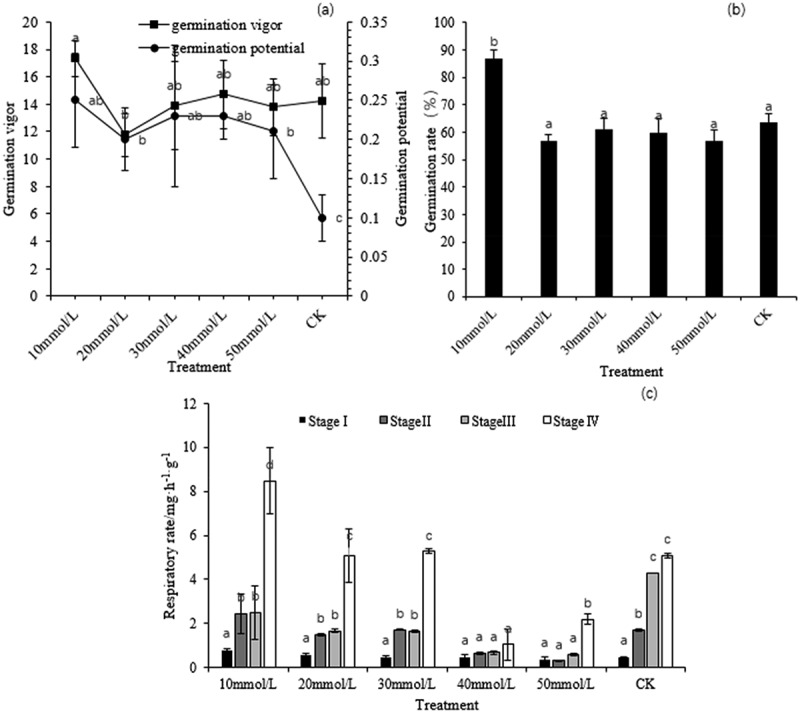
(a) Germination vigor and germination potential variation of seed germination treated with different GA_3_ concentration; (b) Germination rate of seed germination treated with different GA_3_ concentration; (c) Respiration rate of seed germination treated with different GA_3_ concentration. Means with different letters are significantly different between treatment (p < .05).

This study found that the vigor index, germination potential and germination rate of seeds treated with 10 mmol/L GA_3_ were the highest. In addition, CK (Control) had the lowest germination potential compared with the others treated with different GA_3_ concentrations. The vigor index was lowest when the seeds were treated with 20 mmol/L GA_3_. There were no significant differences in germination potential and germination rate between 30 mmol/L, 40 mmol/L GA_3_ treatment groups and CK (shown in Figure 1(a,b)). After GA_3_ treatment, the respiration rate of moso bamboo changed during seed germination. Compared with Stage I, the differences in respiration rate between Stage II and Stage III were narrowed. The respiration rate in Stage IVwas significantly higher than that in other stages under 10 mmol/L, 20 mmol/L,30 mmol/L, 50 mmol/L GA_3_ treatment. The respiration rate at different germination stages was higher after 10 mmol/LGA_3_ treatment and lower after 40 mmol/L and 50 mmol/L GA_3_ treatment (shown in Figure 1(c)).

### 3.2. Ultrastructure of endosperm of germination seed under different GA_3_ treatment

**Figure 2.**
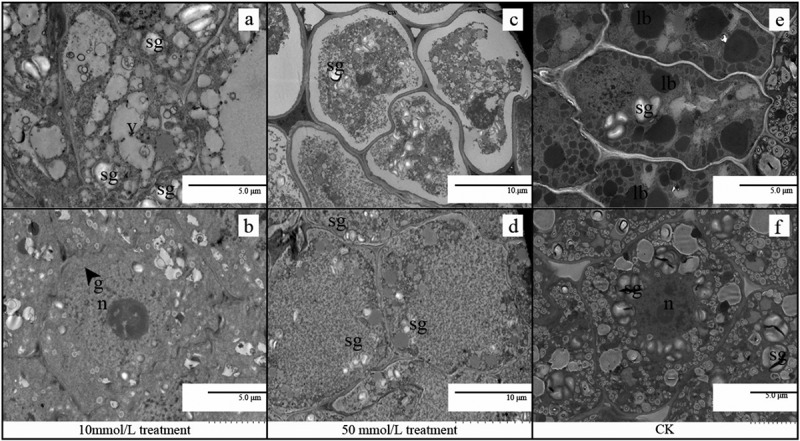
TEM of embryonic cells at stage II with different GA_3_ concentrations treatment(10 mol/L, 50 mol/L, CK: no treatment). (a, b) TEM of embryonic cells at stage II treated with 10 mol/L GA_3_;(c, d) TEM of embryonic cells at stage II treated with 50 mol/L GA_3_; (e, f) TEM of embryonic cells at stage II without treatment. Abbreviations: sg, starch grain; m, mitochondria; g, Golgi apparatus; lb, lipid body; m, mitochondrion; n, nucleus; v, vacuole; mb, multivesicular bodies; ce, cell edge.

The TEM analysis of the ultrastructure revealed the lipid body and starch filled the entire cell of the CK (as shown in Figure 2(e,f)). After 10 mol/L treatment, the starch grains were reduced, and golgi appeared, and many vacuoles with different shapes were formed (shown in Figure 2(a,b)). After 50 mol/L treatment, starch granules and lipid bodies were only distributed in small quantities at the cell edge, and the internal structure of the whole cell was not obvious, and many cytoplasmic walls were separated (as shown in Figure 2(c,d)).

**Figure 3.**
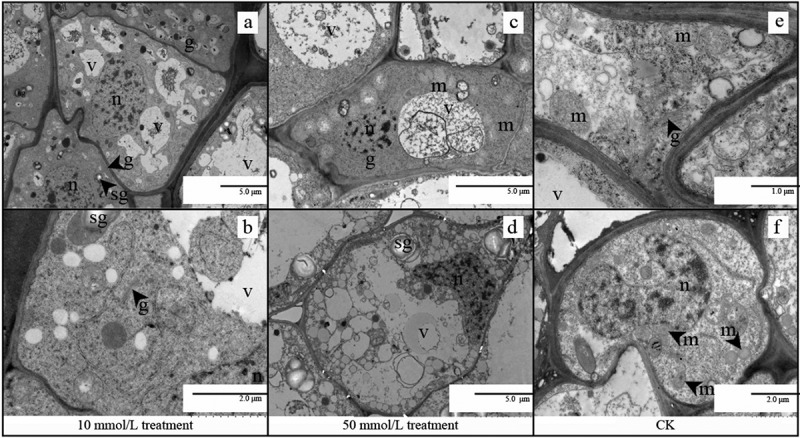
TEM of embryonic cells at stage IIIwith different GA_3_ concentrations treatment (10 mol/L, 50 mol/L, CK: no treatment). (a, b) TEM of embryonic cells at stage III treated with 10 mol/L GA_3_;(c, d) TEM of embryonic cells at stage III treated with 50 mol/L GA_3_; (e, f) TEM of embryonic cells in Stage III without treatment. Abbreviations: sg, starch grain; m, mitochondria; g, Golgi apparatus; lb, lipid body; m, mitochondrion; n, nucleus; v, vacuole; mb, multivesicular bodies.

The number and size of starch grains in seeds treated with 10 mol/L were smaller than those in the untreated group (as shown in Figure 3(a,b)). After 50 mol/L treatment, a few large and irregular vacuoles and minor deposits dominated the germination seeds, and most of the cells had no obvious organelles except nucleus (shown in Figure 3(c,d)).

**Figure 4.**
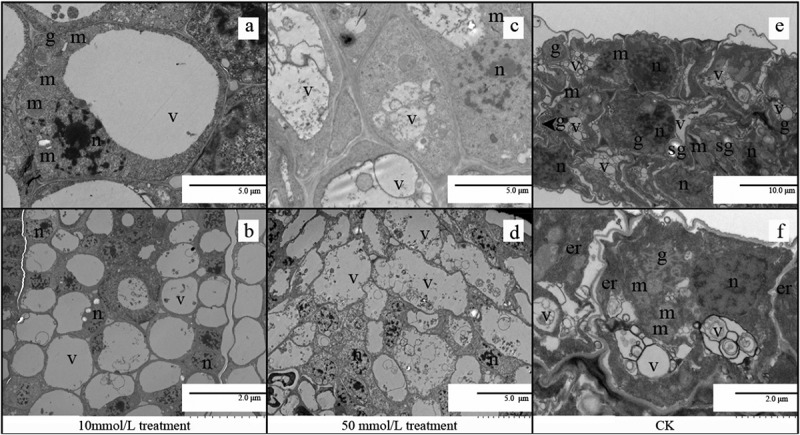
TEM of embryonic cells at stageIV with different GA_3_ concentrations treatment (10 mol/L, 50 mol/L, CK: no treatment). (a, b) TEM of embryonic cells at stage IV treated with 10 mol/L GA_3_;(c, d) TEM of embryonic cells at stage IV treated with 50 mol/L GA_3_; (e, f) TEM of embryonic cells in stage IVwithout treatment. Abbreviations: sg, starch grain; m, mitochondria; g, Golgi apparatus; lb, lipid body; m, mitochondrion; n, nucleus; v, vacuole; mb, multivesicular bodies.

The nucleus, secretory vesicles, many mitochondria and golgi apparatus were observed in CK (shown in Figure 4(e,f)) After the treatment with 10 mol/L GA_3_, the number of mitochondria increased and the storage substances (lipids) of the cells degraded in the germination process in contrast to untreated seeds at Stage IV. Each cell had a large vacuole, and the nuclei of mitochondria and gower were squeezed to the periphery (as shown in Figure 4(a,b)). After the treatment with 50 mol/L, no other organelles were observed in the cotyledon cells of all the seeds apart from the nucleus with the electron-dense nucleolus. Each cell is almost full of fluid, and the internal structure of the cell is chaotic (shown in Figure 4(c,d)).

### 3.3. Transcriptome analysis of DEGs of germination seed with or without GA_3_ treatment

KEGG classification of DEGs were showed in Figure S1, the genes with up-regulated expressions during the germination of seeds without hormone treatment include those related to carbon metabolism, biosynthesis of amino acids, starch and sucrose metabolism, phenylalanine metabolism, plant hormone signal transduction (shown in Figure S1a). The gene number of spliceosome was the most abundant downregulated genes of seeds without hormone treatment (shown in Figure S1c). After GA_3_ treatment, there were still many up-regulated genes, and the number of ribosomal genes increased (shown in Figure S1b). In addition, the hormone treatment increased the number of downregulated genes related to biosynthesis of amino acids, carbon metabolism, cell endocytosis and plant hormone signal transduction (shown in Figure S1d).

The number of DEGs in the germination process of moso bamboo seeds treated with GA_3_ was lower than that of the without treatment. There were 1440 common DEGs and 44194 different DEGs of germination seeds between GA_3_ treatment and without treatment. The top 10 DEGs were obtained by comparing DESeq2_log2FC values, the genes with high differential expression under non-hormone treatment mainly focus on oxidoreductase activity, cytosol, integral component of membrane, vacuolar membrane, RNA binding, and oxidoreductase activity, golgi membrane, NAD+ kinase activity, etc. After GA_3_ treatment, the highly DEGs of moso bamboo seed germination were mainly concentrated in ATP binding, telomere maintenance, nucleus, cytokinesis by cell plate formation, nucleosome and karyogamy, etc (shown in Supplemental Table 1).

**Figure 5.**
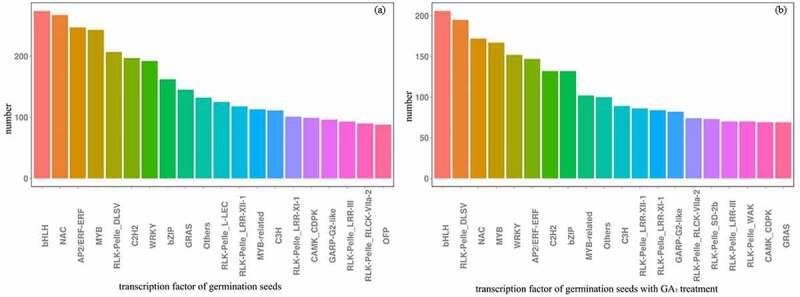
The number of TFs of germination seeds with or without GA_3_ treatment.

There were 6765 TFs of germination seed under non-hormone treatment, while there were 4938 TFs germination seeds under GA_3_ treatment. The number and ratio of RLK-PELle_DLSV and MYB-related TFs were increased by GA_3_ treatment (Figure 5).

### 3.4. Gene coexpression network analysis of germination seeds with or without GA_3_ treatment

**Figure 6.**
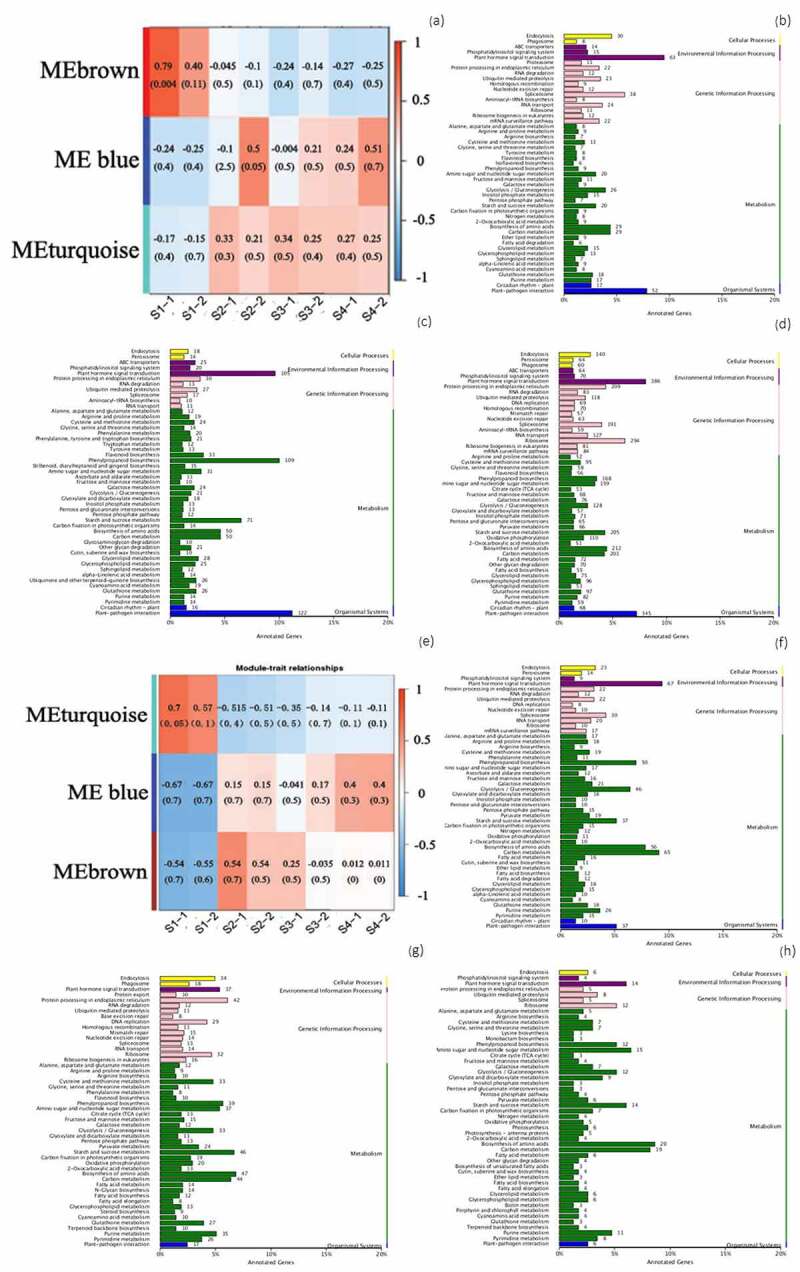
(a) Module trait correlation analysis of genes in seed germination without treatment; (b) The gene number distribution of brown from a; (c) The gene number distribution of blue from a; (d) The gene number distribution of turquoise from a; (e) Module trait correlation analysis of genes in seed germination with 10 mol/L GA_3_ treatment; (f) The gene number distribution of turquoise from e; (g) The gene number distribution of blue from e; (h) The gene number distribution of brown from e.

The gene number distribution of different module showed in Figure 6. In stage II (blue module), contract with non-hormonal treatment, after GA_3_ treatment, the gene number of amino acid biosynthesis and endoplasmic reticulum increased by 0.8% and 1.6%, while the gene number of hormone signal transduction, plant-pathogen interaction, and phenylpropyl biosynthesis decreased by 3%, 5.8%, and 3.2%, respectively (Figure 6(c,g)).In stages III and stages IV (turquoise module), GA_3_ treatment promoted amino acid biosynthesis, glycolysis/gluconeogenesis, and carbon metabolism, with the gene number proportion increased by 1.5%, 1.9%, and 2.4%, respectively, meanwhile the ratio of ribosomal and plant-pathogen interaction gene number decreased by 3.9% and 1.8% respectively, while the number ratio of hormone signal transduction genes did not change (Figure 6(d,h)).

There are hub genes from genes networks which constructed by WGCNA(Figure S2). The hub gene represents the most coexpresssion correlation in each module. Three high coexpression genes PH02Gene41603, PH02Gene05689, PH02Gene05689 in the blue module(represent stage II) of non-hormone treatment are annotated carbohydrate transport and metabolism. However, the genes with high coexpression in turquoise module (represent stages IIand stagesIII) of GA_3_ treatment is PH01000353G0440, whose main functions are phosphatase activity and dephosphorylation. The non-hormone treated turquoise module (represents stages III and stages IV), and its core gene PH02Gene26232 annotated cytoplasmic golgi body, protein transport. The core genes PH01002861G0120, PH01000236G0210 of GA_3_ treatment in blue module (represent stage IV) focus on protein modification, glycosyltransferase-related genes.

### 3.5. Important gene analysis of germination seeds with and without GA_3_ treatment

**Figure 7.**
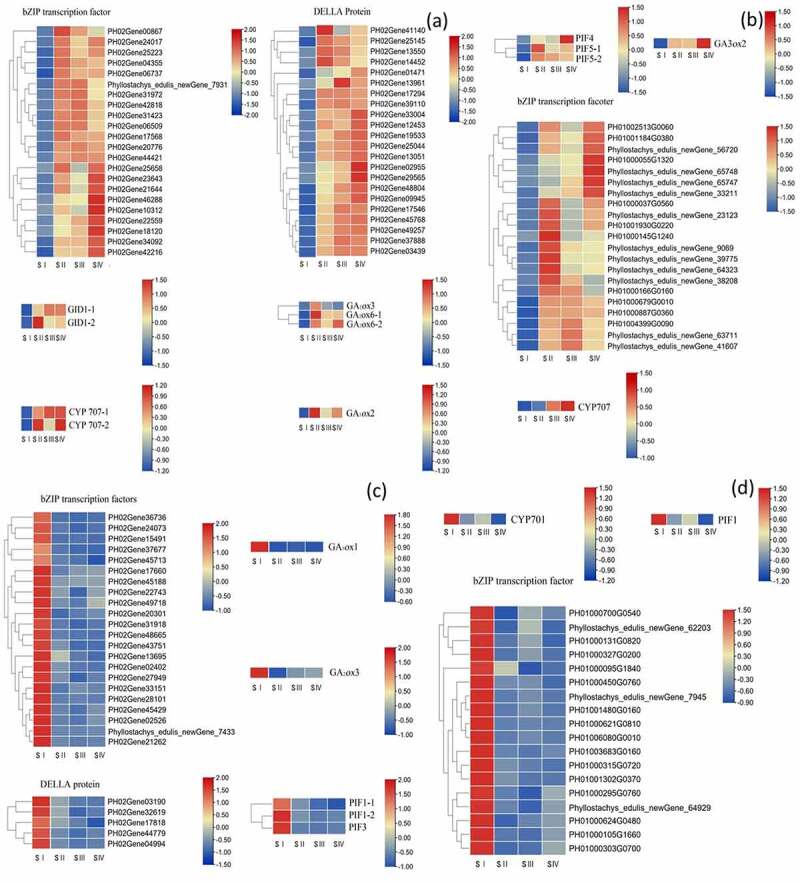
(a) Expression pattern of up-regulated genes during seeds germination without treatment; (b) Expression pattern of up-regulated genes during seeds germination with GA_3_ treatment; (c) Expression pattern of down-regulated genes during seeds germination without treatment; (d) Expression pattern of down-regulated genes during seeds germination with GA_3_ treatment.

The expression levels of important genes homologous to rice genes during the seed germination with and without hormone treatment were analyzed (as shown in Figure 7). After GA_3_ treatment, no DELLA protein gene and GID1 gene homologous to rice genes was expressed (as shown in Figure 7(b,d)). In addition, 21 genes of bZIP transcription factor and homologous to rice genes were up-regulated (as shown in Figure 7(b)), and 18 genes were down-regulated (as shown in Figure 7(d). The up-regulated GA_3_ *ox*2 genes were expressed in treated and untreated samples (as shown in Figure 7 (a,b)), and it had highest expression level at stage IV after hormone treatment (as shown in Figure 7 (b)). But GA_2_ *ox*s was only expressed in the untreated sample (as shown in Figure 7 (a,c)). The same up-regulated PIF4, PIF5 genes (as shown in Figure 7 (b)) and down-regulated PIF1 genes were expressed after hormone treatment (as shown in Figure 7 (c,d)).

### 3.6. Quantitative real-time PCR validation

**Figure 8.**
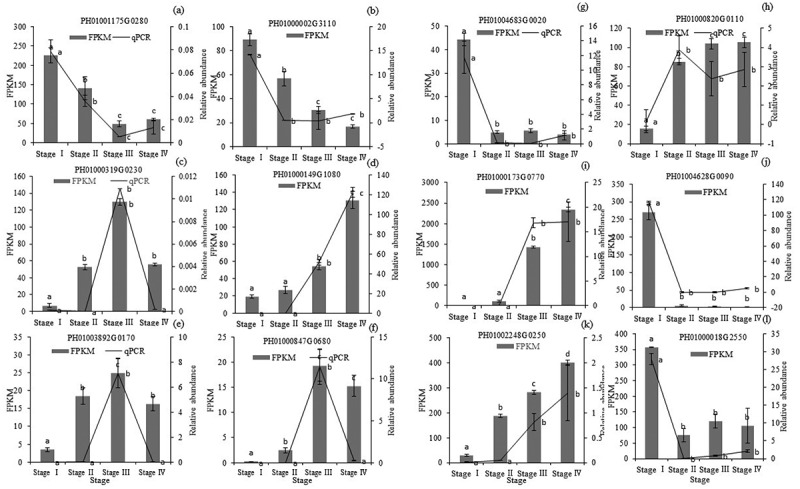
Transcript abundance and relative abundance of genes. (a-f) Genes associated with seed germination with GA_3_ treatment. (g-i) Genes associated with seed germination without treatment. PH01001175G0280: auxin-responsive protein IAA15-like; PH01000002G3110: auxin response factor; PH01000319G0230: glucose-6-phosphate isomerase activity; PH01000149G1080: alpha-amylase activity; PH01003892G0170: flavonoid biosynthetic process; PH01000847G0680: cinnamyl-alcohol dehydrogenase activity; PH01004683G0020: AUX4 protein; PH01000820G0110: aldehyde dehydrogenase (NAD) activity; PH01000173G0770: alpha-amylase activity; PH01004628G0090: alcohol dehydrogenase (NAD) activity; PH01002248G0250: pyruvate, phosphate dikinase; PH01000018G2550: Aspartate aminotransferase, cytoplasmic.

To validate the expression profiles of the RNA-Seq data, twelves DEGs involved in hormonal signal and carbohydrates degradation were selected and determined by quantitative real-time PCR analysis. The FPKM and relative expression of these DEGs had consistent trend. The results exhibited equivalent expression patterns to the RNA-Seq results (Figure 8).

## 4. Discussion/conclusion

### 4.1. Effects of GA_3_ treatment on respiration and ultrastructure of moso bamboo seeds

Compared with that of CK, the respiration rate of moso bamboo seed treated with 10 mmol/L during germination was higher. Lipids, proteins or carbohydrates can serve as respiratory substrates in plants.^27^ Transmission electron microscopy shows that starch and lipid content decreased in seeds treated with 10 mmol/L in stage II(as shown in Figure 2), indicating that GA_3_ accelerated the hydrolysis of starch. Because GA_3_ treatment increased the activity of α -amylase, β -amylase and total amylase in the embryo, then the soluble sugar content of respiratory substrates and the respiration rate of moso bamboo seeds was increased(as shown in Figure 1(c)).

Mitochondria are the main organelles for reactive oxygen species production,^28,29^ and mitochondrial activity increases with respiration that occurs during seed germination, It was observed that more golgi apparatus appeared at stage III(as shown in Figure 3), and more mitochondria appeared at stage IV of seeds treated with 10 mmol/L (as shown in Figure 4), so the seed respiration rate, germination rate and germination potential were the highest under the low concentration treatment(as shown in Figure 1(a)).

The seeds treated with 50 mmol/L were damaged at stage II (as shown in Figure 2), and the development of mitochondria and other organs was affected at stages III and IV(as shown in Figures 3 and 4), and high concentrations of GA_3_ lead to growth inhibition and the destruction of the crucial cellular structure, especially membranes.^30^

The number of vacuoles increased slightly (shown in Figure 2), and more phagocytic substances were formed in moso bamboo seeds at different germination stages regardless of low or high GA_3_ concentration treatment, indicating GA_3_ may induce faster vacuolization in embryo. A major function of the endocytic pathway is to sort internalized macro-molecules and membrane proteins, leading to the premature death of the embryo and cotyledons.^31^ A decline in respiration rate may be associated with cellular endocytosis and autophagy,^32^ and further study is needed.

### 4.2. The molecular mechanisms of seed germination under hormone treatment

Gibberellin (GA_3_) is an important plant growth regulator that can cause various physiological changes and thus profoundly influences seed germination through the signal transduction pathway and the regulation of a series of proteins and enzymes metabolism.^33,34^ The molecular mechanism of GA_3_ regulating signal transduction pathways and hormone synthesis could be preliminarily explored through transcriptome sequencing and gene co-expression analysis of DEGs during moso bamboo seed germination.

The results of DEGs expression and co-expression module of GA_3_ treated samples were related to non-hormone-treated samples, such as starch and sugar metabolism play a very important role in seed development and embryo viability, whether the seed is treated with GA_3_ or not.However, there are many differences between them. After hormone treatment, the amino acid synthesis, carbohydrate transport and metabolism accelerated (shown in Figure 6(g,h), Figure S1d). And the core genes are related to dephosphorylation and glycosyltransferase(Figure S2b), because phosphorylation can play a direct role in the regulation of seed dormancy and germination, or indirectly regulate the biological processes such as seed dormancy and germination and stress response by affecting the hormone signal transduction such as ABA(Abscisic Acid)/GA.^35^At the same time, glycosyltransferase can promote seed germination under stress environment,^36^ suggesting that GA_3_ treatment promotes the these two genes expression in moso bamboo during seeds germination.The number and ratio of receptor-like kinases-PELle_ DLSV (RLK-PELle_DLSV) and MYB-related TFs were increased by GA_3_ treatment(shown in Figure 5), because MYB was closely related to the plant hormone response pathway, RLKs is a special class of kinases that participate in phosphorylation as specific transmembrane proteins, which was also be highlighted in this study.

No Della protein gene and GID1 gene homologous to rice was detected after hormone treatment. The GA3 is an activator of seed germination and antagonizes the inhibition of ABA on seed germination by degrading DELLA proteins and activating downstream GA signals ^37–39^. The loss of DELLA function up-regulated the TFs, such as Leafy Cotyledon1, (LEC1), LEC2, FUSCA3 (FUS3), ABSCISIC ACID INSENSITIVE3 (ABI3) and WRINKLED1 (WRI1), which regulate embryonic and seed development.^40^ In addition, GA promotes the interaction between GID1 and DELLA by binding to a GID1 receptor to degrade DELLA proteins and finally removes their restriction on seed germination.^41–45^

The number and expression of PIFs changed after hormone treatment, because the interaction between DELLA and PIFs suppresses the binding of the latter to target promoters and thus inhibits their activity. Although DELLAs lack a DNA binding domain, they possess transactivation properties,^46^ and several studies have shown that DELLAs can act as coregulators and directly regulate gene expressions when interacting with transcription factors.^46–48^ It is speculated that under the effect of exogenous GA_3_, DELLA interacts with PIF1, genes encoding PIF4 and PIF5 downstream of GA signal pathway are still up-regulated, and the GA signal transduction pathway is enhanced to promote the seed germination of moso bamboo.

The genes of GA_20_*ox*1, GA_20_*ox*2, GA_20_*ox*3 and GA_2_*ox*1 are closely related to the synthesis and decomposition of GA_3_.^49^ The GA_2_*ox*s were not detected after hormone treatment because GA homeostasis is regulated by a negative feedback loop in moso bamboo, and GA_2_*ox*2 inhibits the germination by catalyzing GA deactivation,^50^ and GA_2_*ox* is normally regulated by environmental signals, making endogenous GAs extremely sensitive to environmental changes. The GA_20_*ox* and GA_3_*ox* are key enzymes that regulate the synthesis of active GA,^51^ and genes involved in GA biosynthesis (GA_3_*ox*2, GA_20_*ox*2) promote seed germination by increasing GA content.^52^ The GA_3_*ox*s genes were presented in both hormonal and non-hormonal treatments. In addition, hormone treatment can promote the number of endocytosis genes (as shown in Figure 5(d)) in the seed ultrastructure, which was verified in this study. In addition, protein phosphate2C negatively regulates seed dormancy, protein phosphate2C increase the GA response gene (Cycloidea/Proliferating,CP1 and expansin1, EXP1) and GA (GA_3_*ox*1 and GA_3_*ox*2) synthetic gene expression, inhibition of ABA signals and activate the GA, and then the seeds break dormancy and the germination transition.^53^ We speculate that the co-expression of core genes in this study is related to the expression of important gene GA_3_*ox*s.

## Supplementary Material

Supplemental Material
