# Supplementary figures and images for: Ultrastructure change and transcriptome analysis of GA3 treatment on seed germination of moso bamboo(Phyllostachys edulis)
Source: Plant Signal Behav. 2022 Jul 7;17(1):2091305. doi: 10.1080/15592324.2022.2091305 (PMC9272834; doi:10.1080/15592324.2022.2091305)

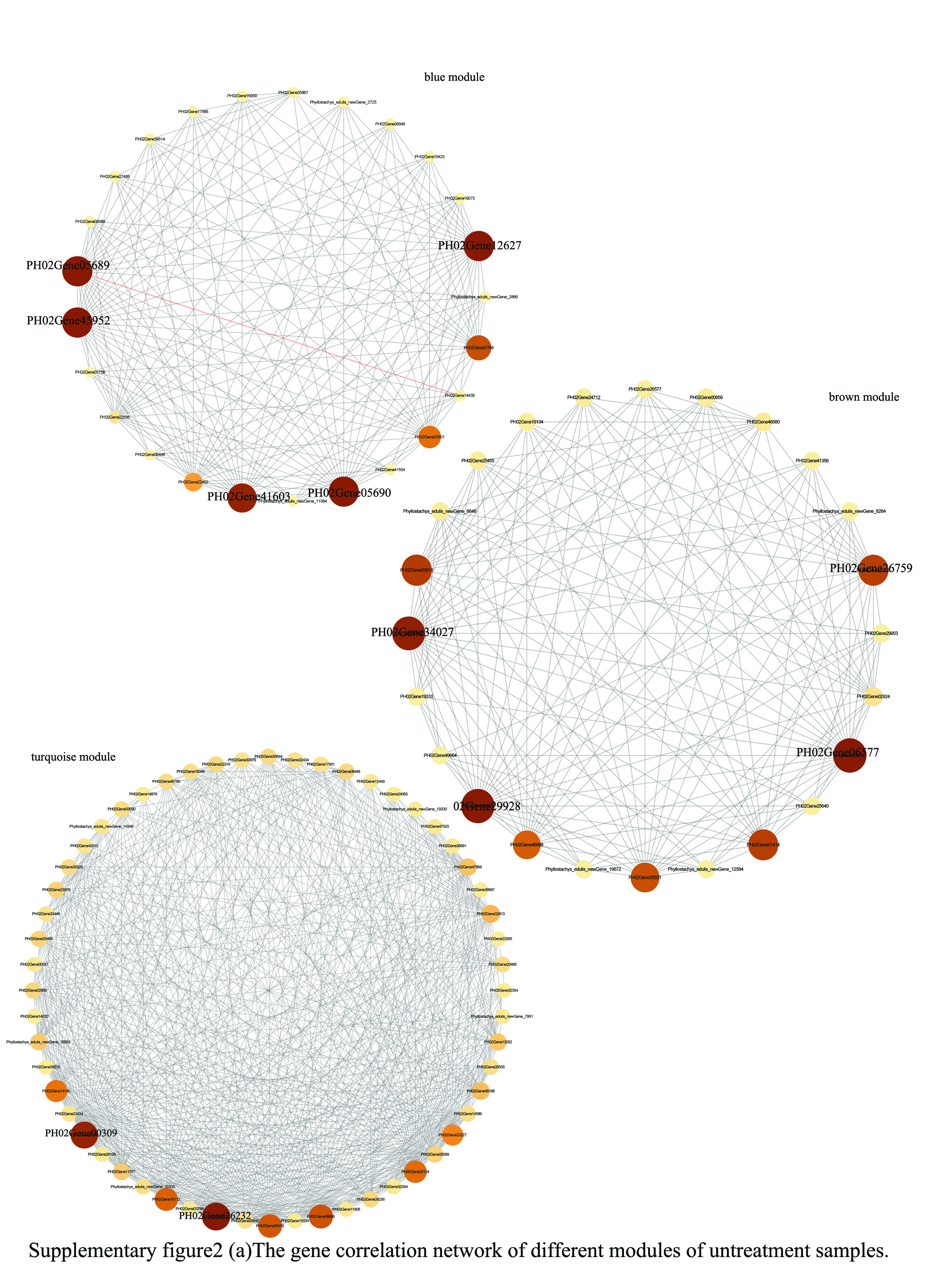

Supplement: Supplemental Material [file KPSB_A_2091305_SM1308.zip › Supplementary figure 2(a).jpg]

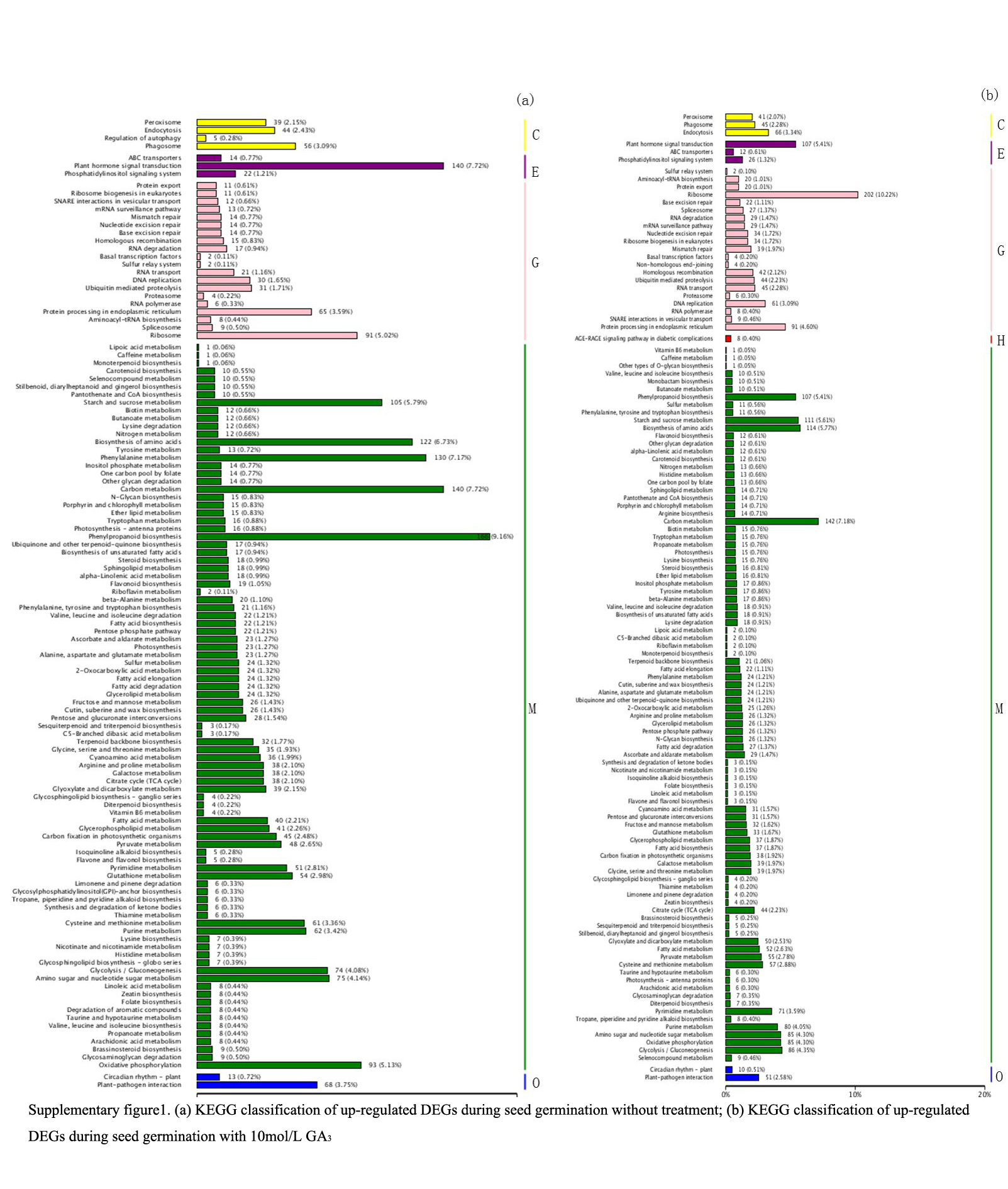

Supplement: Supplemental Material [file KPSB_A_2091305_SM1308.zip › Supplementary figure1(a)(b).jpg]

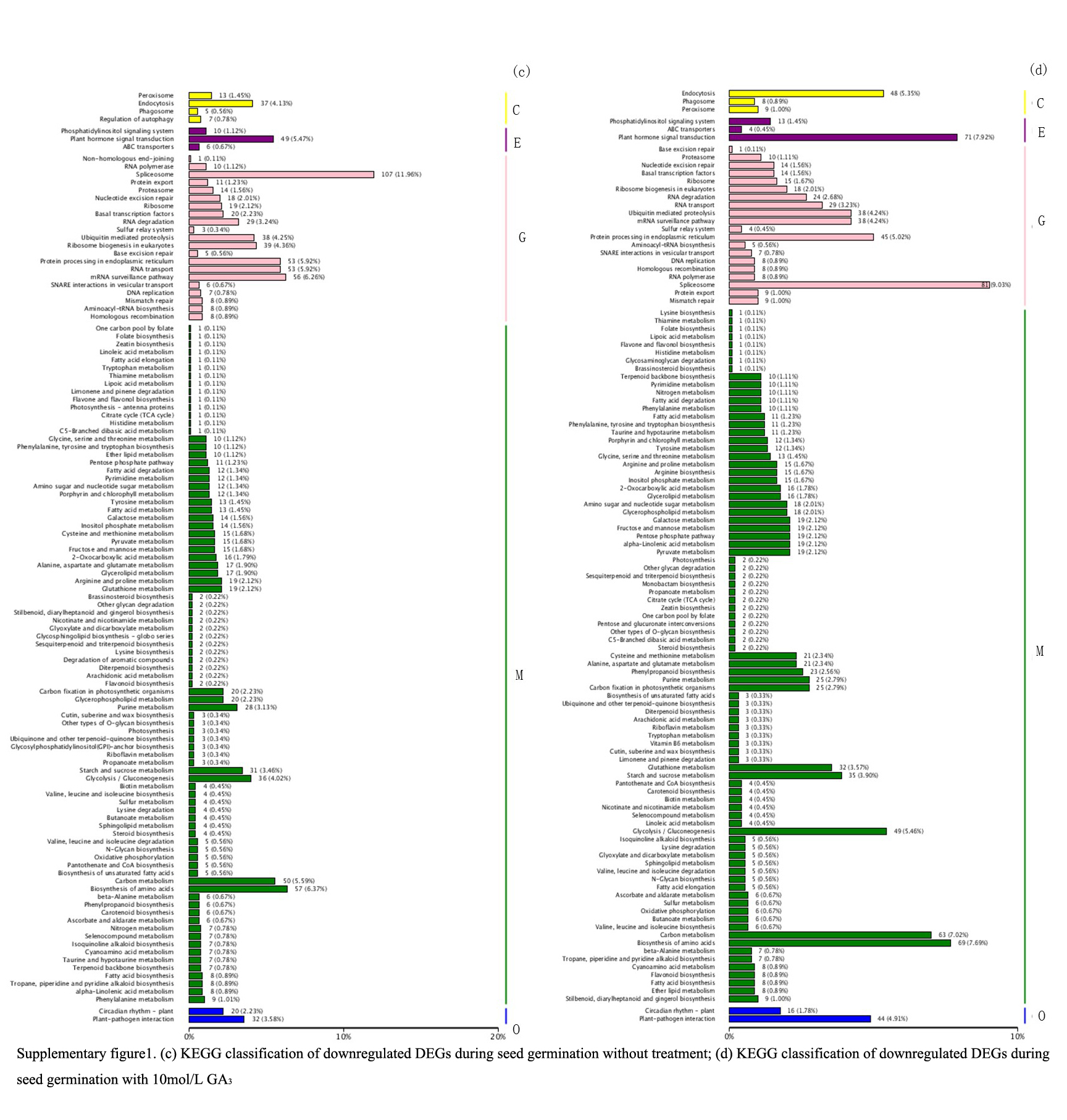

Supplement: Supplemental Material [file KPSB_A_2091305_SM1308.zip › Supplementary figure1(c)(d).jpg]

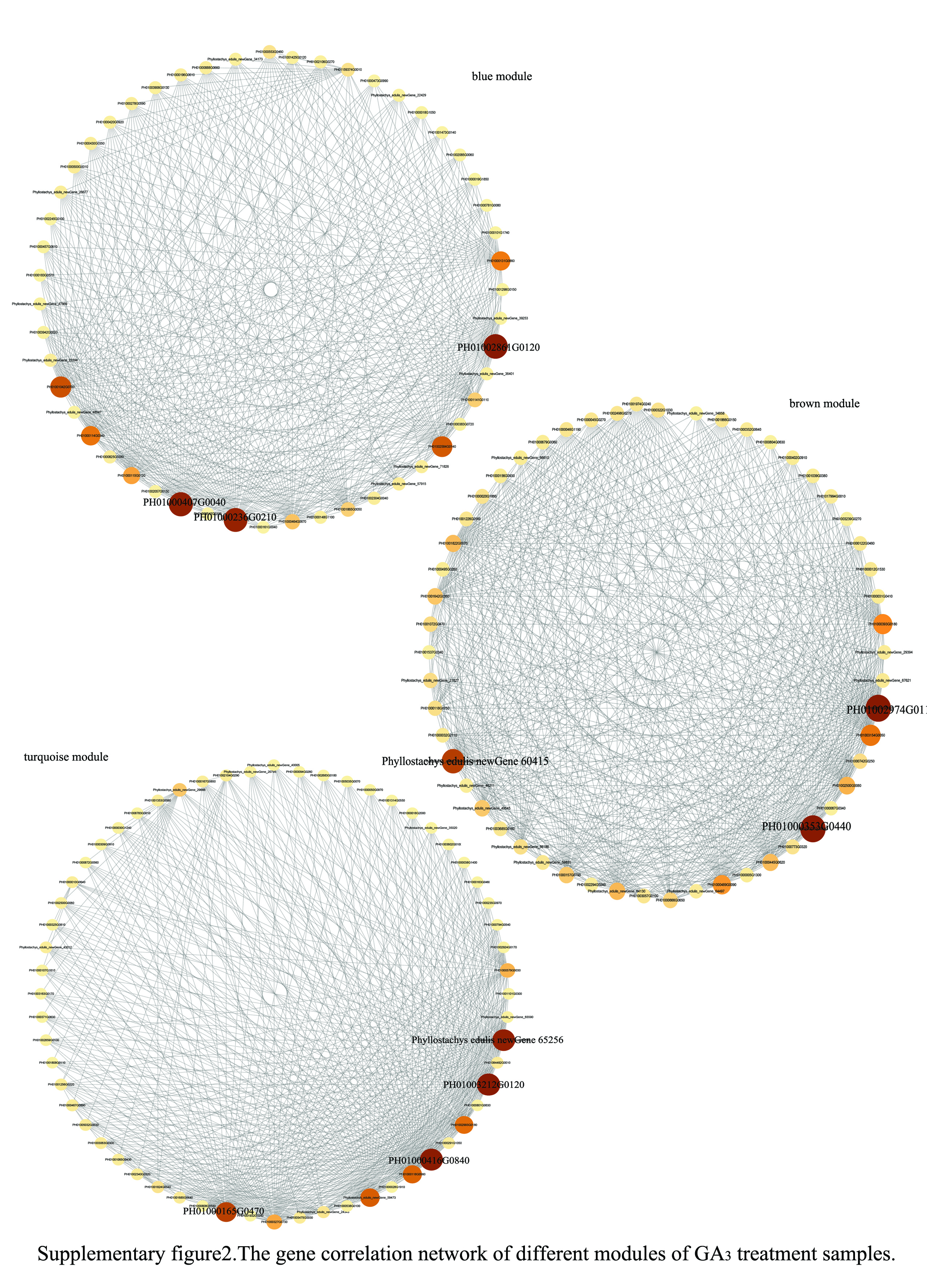

Supplement: Supplemental Material [file KPSB_A_2091305_SM1308.zip › Supplementary figure2(b).jpg]
